# CT-based radiomics analysis to predict local progression of recurrent colorectal liver metastases after microwave ablation

**DOI:** 10.1097/MD.0000000000036586

**Published:** 2023-12-29

**Authors:** Hao Hu, Jia Chang Chi, Bo Zhai, Jin He Guo

**Affiliations:** a Center of Interventional Radiology & Vascular Surgery, Department of Radiology, Zhongda Hospital, Medical School, Southeast University, Nanjing, China; b Basic Medicine Research and Innovation Center of Ministry of Education, Zhongda Hospital, Southeast University, Nanjing, China; c Department of Interventional Oncology, Renji Hospital, School of Medicine, Shanghai Jiao Tong University, Shanghai, China.

**Keywords:** colorectal liver metastases, local progression, microwave ablation, radiomics

## Abstract

The objective of this study is to establish and validate a radiomics nomogram for prediction of local tumor progression (LTP) after microwave ablation (MWA) for recurrent colorectal liver metastases (CRLM) after hepatic resection. We included 318 consecutive recurrent CRLM patients (216 of training while 102 of validation cohort) with contrast-enhanced computerized tomography images treated with MWA between January 2014 and October 2018. Support vector machine-generated radiomics signature was incorporated together with clinical information to establish a radiomics nomogram. Our constructed radiomics signature including 15 features (first-order intensity statistics features, shape and size-based features, gray level size zone/dependence matrix features) performed well in assessing LTP for both cohorts. With regard to its predictive performance, its C-index was 0.912, compared to the clinical or radiomics models only (c-statistic 0.89 and 0.75, respectively) in the training cohort. In the validation cohort, the radiomics nomogram had better performance (area under the curve = 0.89) compared to the radiomics and clinical models (0.85 and 0.69). According to decision curve analysis, our as-constructed radiomics nomogram showed high clinical utility. As revealed by survival analysis, LTP showed worse progression-free survival (3-year progression-free survival 42.6% vs 78.4%, *P* < .01). High-risk patients identified using this radiomics signature exhibited worse LTP compared with low-risk patients (3-year LTP 80.2% vs 48.6%, *P* < .01). A radiomics-based nomogram of pre-ablation computerized tomography imaging may be the precious biomarker model for predicting LTP and personalized risk stratification for recurrent CRLM after hepatic resection treated by MWA.

## 1. Introduction

Colorectal cancer liver metastasis (CRLM) shows the highest prevalence among metastatic lesions in the liver, which is observed in about 50% of colorectal cancer patients in the disease course.^[1]^ Hepatic resection remains the first treatment option for CRLM, and the 5-year overall survival (OS) rate is as high as 50% to 60%, however, only <1/5 of patients are the candidates for resection when they are first diagnosed^[2,3]^ because of tumor number, functional liver reserve, and unfavorable location close to vital structures. Additionally, 50% to 70% of patients receiving initial curative treatment may suffer from intrahepatic relapse, and repeated hepatic resection has been recognized as the preferred treatment option in these patients.^[4,5]^ Nonetheless, it induces an increased complication rate because of the technical challenges (adhesions from prior resection) and restricted future liver remnant.^[6]^

Percutaneous thermal ablation is widely applied for hepatic malignancies.^[7]^ As an alternative to surgery, thermal ablation, including microwave ablation (MWA) can enhance the survival of patients with oligometastasis and restrict symptoms and complications because of liver metastases.^[8]^ Numerous studies have shown that the use of thermal ablation can provide comparable prognosis to surgical resection for resectable CRLM.^[9]^ Indeed, MWA can achieve a favorable local control rate similar to surgical resection, and it is advantageous because repeated ablations do not significantly affect liver function.^[10,11]^ The 2021 National Comprehensive Cancer Network guidelines indicated that ablation technique for colorectal liver metastases can be used with surgery or alone as the local curative treatment for selected small CRLM and resectable visible diseases.^[12]^ It is important to identify early predicting factors for local recurrence following MWA, since this may affect OS. Therefore, identifying high-risk local tumor progression (LTP) patients early is important for the optimal guidance of the planning of ablation and follow-up after ablation, for instance, alternative and further treatments are applicable for high-risk patients for the sake of reducing liver progression risk, subsequent metastasis spreading, and finally improving OS.^[13,14]^

Radiomics has recently emerged as the imaging analysis modality based on various statistical analytical approaches or datamining algorithms to analyze high-throughput imaging features for obtaining information for prognosis prediction. Through constructing suitable models by incorporating refined features, radiomics can successfully assess and predict some challenging tasks.^[15,16]^ Such methods are more and more utilized to characterize, grade, and assess treatment response of tumors, mainly in liver. At present, radiomics is increasingly applied in highly sensitively identifying liver metastases compared with MWA or early response patterns following MWA.^[17,18]^

In a number of studies, the clinical risk score models can be applied in predicting OS and disease-free survival following thermal ablation of CRLMs,^[18,19]^ nonetheless, few radiomics-based models combined with clinical indicators are adopted for predicting intrahepatic progression-free survival (PFS) following MWA due to recurrent CRLMs. This work focused on investigating whether the radiomics-based nomogram could be used to predict LTP after MWA for recurrent CRLM following hepatic resection. Besides, the nomogram was correlated with clinical factors and survival.

## 2. Materials and methods

### 2.1. Study participants, endpoints, and follow-up

The present retrospective study, patients received standard care in one medical center. The study protocol gained approval from the institutional review board. Our institutional database was searched and altogether 652 patients for recurrent CRLM following hepatic resection treated with MWA were discovered between January 2014 and May 2018. Patients below were included: (a) diagnosis of colorectal cancer made through histopathology, (b) recurrent CRLM after hepatic resection, (c) MWA for at least 1 liver metastasis (at most 5 lesions in each patient), with lesion diameter of ≤3 cm, (d) available high-quality upper abdomen contrast-enhanced computerized tomography (CT) at the portal venous phase <8 weeks before ablation., (e) those achieving tumoral complete response following one individual ablation treatment, and (f) MWA that gained approval from multidisciplinary board of our comprehensive cancer center. Exclusion criteria were as follows: (a) those achieving tumoral complete response following one individual ablation treatment, (b) those with underlying hepatopathies like steatosis and cirrhosis, (c) those with metastatic lesions of unclear origin, and (d) those with underlying (unresectable) extra-hepatic disorder. We included altogether 318 patients in this study and classified them as training (n = 216) or validation group (n = 102) at random. CRLM patients have been confirmed through imaging diagnosis, and a portion of patients underwent preoperative biopsy of liver tumor due to unclear diagnosis (n = 68). We deemed LTP to be tumor focus occurrence on the ablation zone border after one or more contrast-enhanced follow-up CT or MRI reported no viable tissue within target tumor or neighboring ablation margin.^[20]^ Follow-up examinations included liver imaging examinations at 4-week after ablation, and additional 3 examinations within 1 year. At 1 year later, patients received regular follow-up examination of colorectal cancer in line with national guidelines, including carcinoma embryonic antigen (at 3-6-month intervals) and liver imaging (at 6-month intervals within initial 2 years, and later 1-year intervals till 5 years following primary tumor resection).

### 2.2. Microwave ablation

MWA was ultrasound-guided ablation in the operating room or percutaneous CT-guided in the CT-room. The 2450-MHz MWA system was used (Vision Medical, Nanjing, China), which was equipped with 1 microwave generator (power output, 1–100 W), 1 cooled-shaft antenna, and 1 flexible coaxial cable (Vision Medical, Nanjing, China). The antenna of the dual channel was 1.9 mm and 10 to 18 cm in diameter and length, separately (15 G), and the shaft was cooled persistently through circulating cold saline through the antenna with the peristaltic pump. MWA procedure conducted at our institution was depicted below. Lesions ≤2.0 cm in diameter was treated with 1 individual antenna and 1 individual insertion, those 2.0 to 3.0 cm in diameter were treated with 1 antenna and 1 to 2 insertions; and those >3.0 cm were treated with 2 antennas together with several insertions. Two antennas were separated for 1.0 to 1.5 cm. Energy output is regulated at 60 to 80 W in 5 to 15 minutes, and the ablation area is expanded as far as possible to cover the lesion and surrounding hepatic parenchyma by at least 5 mm. At the end of this process, ablation of needle track was conducted for preventing tumor seeding or hemorrhage. One radiologist with more than 10-year experience of liver cancer ablation was responsible for completing all procedures. For the sake of achieving expected ablation margin, we placed an electrode for penetrating the lesion center under the guidance of Ultrasound/CT. Dynamic contrast-enhanced CT was carried out within 1 week post-MWA to evaluate safety margin for ablation zone. Minimal ablation margin was evaluated on those 3 orthogonal planes according to previous description based on initial cross-sectional contrast-enhanced imaging examination post-ablation.^[21]^ Besides, minimal ablation margin attained in each 3D axis was adopted for categorizing ablated CRLM into having ≤10 mm or >10 mm of minimal ablation margin, following the recent expert recommendation.^[22]^

### 2.3. Image collection and segmentation

The upper-abdomen portal-venous-phase CT image was obtained 1 month prior to ablation. Thereafter, 3D tumor lesion segmentation was completed by 1 radiologist who had 10-year experience with ITK-SNAP software.^[23]^ Those interested regions were delineated manually at the enhanced portal venous phase that covered the entire lesion. Later, 1 senior radiologist who had 20-year experience was responsible for validating segmentation results. Afterwards, test-retests were carried out on 30 tumor samples selected at random, for the sake of testing whether our features extracted by repeated segmentation were reproducible and excluding features whose intraclass correlation coefficients were <0.8.

### 2.4. Feature extraction, scaling, and selection

Pyradiomics package (version 3.7) was applied in 3D radiomics feature extraction. First of all, image normalization should be completed for reducing differences among CT scan protocols. The process is as follows: First, the voxel size should be resampling to 3 × 3 × 3 mm^3^ to make it isotropic to reduce the variation resulting from heterogeneities of scanning equipment and protocols or different focal sizes of patients. Standardize voxel spacing to minimize the dependence of radiomic features on image voxel size. Ensure that the actual physical size represented by each voxel is consistent to reduce the impact of individual differences. Second, voxel intensities were discretized by using the 25-HU fixed bin width, thereby reducing image noise and standardizing the intensity, achieving stable intensity resolution in different images. Third, the image is normalized: the signal strength is normalized to 1 to 500 HU, so as to reduce the difference in the signal strength of images collected by different machines. Fourth, *Z*-score is used to standardize the gray value of the image to reduce the influence of image parameter inconsistency on the variation of radiomic features.

Radiomics features were extracted form original images, as well as from image transformations. Filters applied to images included Laplacian of Gaussian, wavelets, exponential, gradient, square, square-root, and logarithm. In general, transformation methods allowing for better enhancement of edges can provide information about the spatial location of image features or it can remove noise. Radiomics features can be divided in different feature classes: first-order intensity statistics features, two-dimensional (2D) features that depicted region of interest shape and size, gray-level co-occurrence matrix (GLCM), gray level size zone matrix, gray-level run-length matrix, neighboring gray tone difference matrix, and gray level dependence matrix. Besides, we used 14 filters, such as exponential, gradient, square, square root, logarithm, lbp2D, wavelet-HLH, wavelet-HLL, wavelet-LHL, wavelet-LLL, wavelet-LHH, wavelet-LLH, wavelet-HHL, and wavelet-HHH, on original images and obtained derived images in every patient.

Feature scaling was performed below: First, mean values were subtracted from every feature. Second, every feature value was divided by the corresponding standard deviation. Features of high-dimensionality probably result in inefficient computation or overfitting. Consequently, the least absolute shrinkage and selection operator (LASSO) approach, which allows for dimensionality reduction, was used for selecting informative features. First, optimal parameter *λ* was obtained through 10-fold cross-validation under 1000 iterations. Thereafter, we used LASSO approach on the basis of optimal parameter *λ* for calculating feature coefficients, and chose features of nonzero coefficients.

### 2.5. Machine learning-based model construction

We divided the whole dataset as the training and the independent validation sets at random. Three predictive models were designed. The radiomic model was trained on radiomic features while the clinical model used clinical features only. In addition, the combined model was trained including clinical and radiomic features.

To build the predictive models, a machine learning pipeline with 3 steps was designed. In the first step, features were standardized with zero mean and unit variance. For the second step of feature selection, a wrapper method was used to find the best set of features. The wrapper model approach uses the methods of classification itself to measure the importance of a feature set. In the third step, support vector machine (SVM) was used for classification. SVM, as the supervised learning approach, is extensively utilized in regression analysis and statistical classification. It allows for mapping the vector to the higher-dimensional space, where the maximal interval hyperplane can be generated. Consequently, such higher-dimensional space-derived vectors can attain higher classification efficiency. A hyperparameter in the machine learning model can be tunable, which must be initialized prior to model training and is important for model performance. Bayesian hyperparameter optimization, which is the iterative search process using simpler machine learning algorithms for finding hyperparameter combinations with the best performance, was used. This process was carried out in training set through fivefold cross-validation under 1000 iterations for identifying the optimal hyperparameters.

Model performance was evaluated using accuracy (ACC), positive predictive value (PPV), negative predictive value (NPV), sensitivity, specificity and area under receiver operating characteristic curve (AUC). Moreover, model performances were displayed and compared by drawing receiver operating characteristic curves.

### 2.6. Clinical and radiomics nomogram development

**Figure 1.**
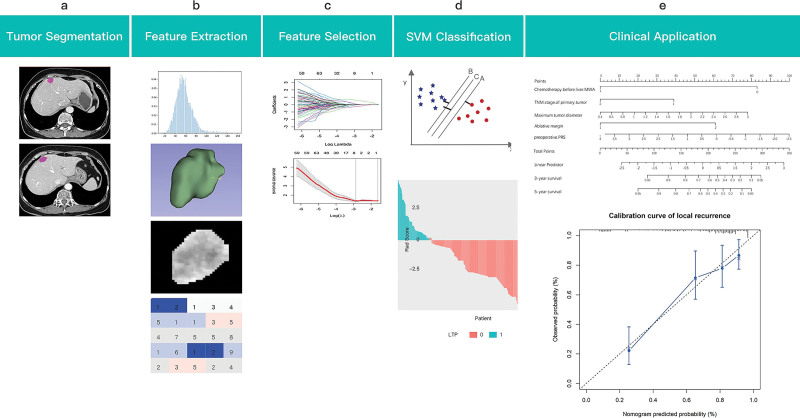
Radiomics processing pipeline.

Multivariable logistic regression model-based nomograms are shown in Figure 1. The clinical parameters used in the clinical model include the following: age; gender; tumor node metastasis (TNM) stage; initial tumor location; RAS mutated; ablative margin; clinical risk score; interval between primary resection and MWA; chemotherapy before liver MWA; adjuvant chemotherapy after liver MWA; carcinoma embryonic antigen level; CA19-9 level; no. of target liver metastases; maximum tumor diameter and tumor location. Radscore based on CT images was also used to construct the radiomics nomogram. Backward stepwise logistic regression was terminated by applying likelihood ratio test with Akaike’s information criterion as the criterion. Additionally, discrimination performance was quantified with Harrell C-index, AUC, ACC, PPV, and NPV. Model utility was evaluated by decision curve analysis.

### 2.7. Statistical analysis

All statistical analyses were performed using the SPSS (version 20, Chicago, IL) and R software (version 3.4.1, Boston, MA). Chi-squared test was used to analyze the categorical variables, and the *t* test was applied to analyze the continuous variables with a normal distribution, and the Mann–Whitney *U* test was used for an abnormal or unknown distribution. A 2-tailed *P* value <.05 was considered statistically significant. R software was used to build and evaluate the prediction model. The “glmnet” package was used for LASSO logistic regression analyses, and the “Bglm” function was used for the univariate and multivariate logistic regression analyses. The “Hmisc” package, “pROC” package, “Calibration Curves” package, and “Decision Curve” package were used for nomogram, receiver operating characteristics, calibration curves, and DCA analysis respectively. Statistical methods used in radiomics and machine learning have been described in Radiomics analysis section.

## 3. Results

### 3.1. Characteristics of the study cohorts

Table 1 shows detailed baseline characteristics of all patients; clinical characteristics did not differ between the training and validation cohorts. The median duration of follow-up was 36.8 months (interquartile range, 25.6–50.2 months) for the training cohort and 38.4 months (interquartile range, 26.7–51.8 months) for the validation cohort. Median duration from pre-MWA CT to ablation was 31 (2–58) days for the training cohort and 29 (2–56) days for validation cohort. LTP was comparable in both cohorts (*P* = .39); 58/216 (26.9%) patients had LTP in 66/315 (21.0%) lesions for the training cohort, and 27/102 (26.5%) patients of validation cohort suffered LTP within 32/140 (22.9%) lesions.

**Table 1:** Baseline characteristics of the study population.

Characteristics	Training cohort (n = 216)	Validation cohort (n = 102)	*P* value
Age (yr)	62 ± 11	61 ± 10	.892
Female n (%)	74 (34.2)	35 (34.3)	.545
Initial tumor location (rectum/colon)	80 (37.0)/136 (63.0)	40 (39.2)/62 (60.8)	.4
RAS mutated	81 (37.5)	40 (39.2)	.431
TNM stage of primary tumor (I–II/III–IV)	69 (32)/147 (68)	31 (30.4)/71 (69.6)	.443
Intravascular tumor thrombus	34 (15.7)	17 (16.7)	.476
Perineural invasion	28 (13.0)	15 (14.7)	.396
Interval between primary resection and MWA	32.8 ± 29.4	34.5 ± 26.3	.326
Chemotherapy before liver MWA	60 (27.8)	27 (26.5)	.459
Adjuvant chemotherapy after liver MWA	50 (23.1)	26 (25.5)	.373
CEA level (ng/mL) (≥5 ng/mL)	72 (33.3)	31 (30.4)	.348
CA19-9 level (ng/mL) (≥27 U/mL)	40 (18.5)	17 (16.7)	.408
Clinical risk score 3–4	82 (38.0)	40 (39.2)	.446
FDG uptake of hepatic metastasis (positive)	171 (79.2)	82 (80.4)	.463
No. of target liver metastases (multiple)	66 (30.6)	34 (33.3)	.354
Maximum tumor diameter (cm)	1.4 ± 0.6	1.3 ± 0.5	.528
Tumor location (close to a main vessel or bile duct)	39 (18.1)	17 (16.7)	.447
Minimal ablative margin (>10 mm)	186 (86.1)	90 (88.2)	.568

CEA = carcinoma embryonic antigen, FDG = fluorodeoxyglucose, MWA = microwave ablation, TNM = tumor node metastasis.

### 3.2. Establishment of CT-based radiomics signature

A total of 1642 imaging features were finally calculated for each patient from the extracted tumor region of portal venous phase CT image. After ranking by selection frequency of 34 features (>50% selection probability), 15 with optimal performance were selected in training models. Classification accuracy and the AUC value upon repeated fivefold cross-validation were 85% and 0.84, separately (Table 2). The AUC, ACC, PPV, NPV, sensitivity, and specificity of the SVM model in the training cohort were 0.89, 92%, 91%, 90%, 70%, and 58% separately, and those were 0.85, 88%, 79%, 89%, 68%, and 61%, respectively, in validation cohort (Table 2).

**Table 2:** Performance of SVM classification, clinical model, and radiomics-based nomogram.

Metrics	Radiomics signature		Clinical model		Radiomics-based nomogram	
	Primary cohort	Validation cohort	Primary cohort	Validation cohort	Primary cohort	Validation cohort
AUC	0.89	0.85	0.75	0.69	0.95	0.89
ACC	92%	88%	77%	70%	96%	85%
PPV	91%	79%	71%	64%	95%	94%
NPV	90%	89%	80%	68.%	91%	88%
Sensitivity	70%	68%	44%	42%	89%	86%
Specificity	58%	61%	72%	70%	86%	89%

ACC = accuracy, AUC = area under the curve, NPV = negative predictive value, PPV = positive predictive value.

### 3.3. Establishment of clinical and radiomics nomogram

**Figure 2.**
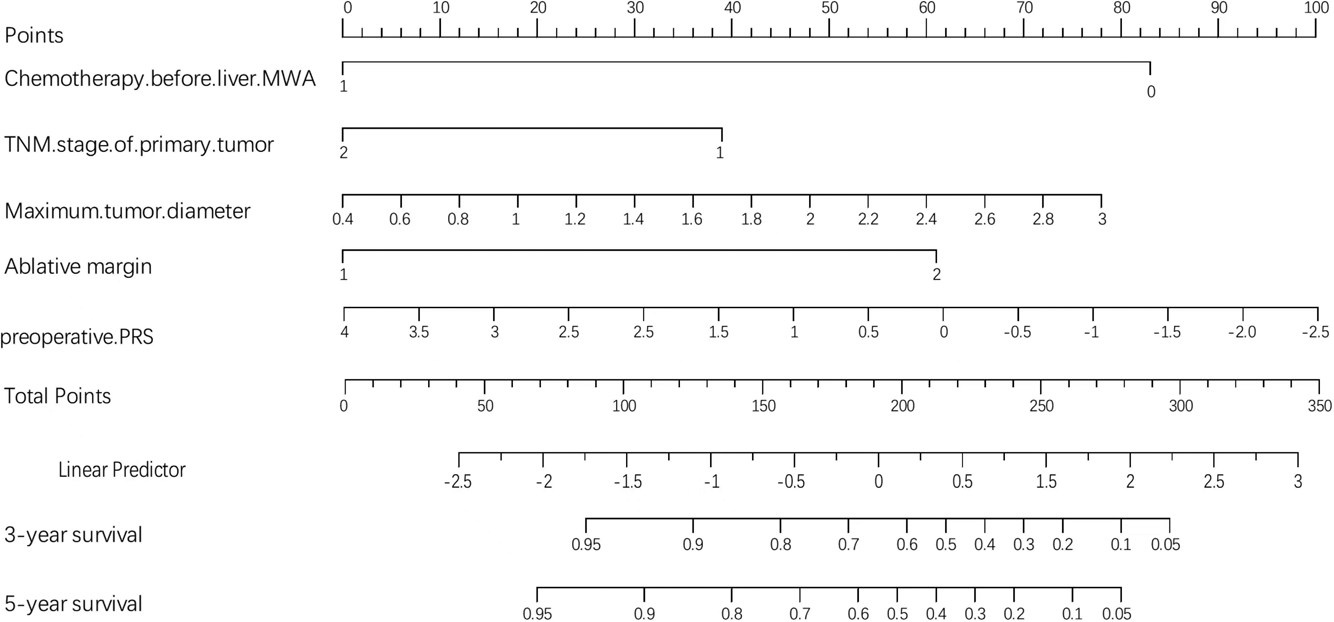
A predictive nomogram combining radiomics with clinical features to predict local recurrence risk in patients with recurrent CRLM following resection after MWA. CRLM = colorectal liver metastases, MWA = microwave ablation.

**Figure 3.**
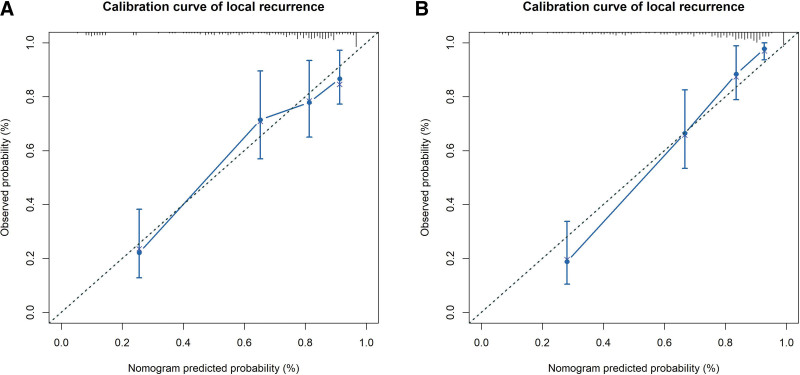
Calibration curves of the preoperative nomogram on the training (A) and validation (B) cohorts.

Adjuvant chemotherapy before liver MWA, maximum tumor diameter, ablative margin, and TNM stage of primary tumor independently predicted the risk of LTP (*P* < .05). The C-index values of the clinical nomogram constructed by incorporating the above 4 factors were 0.698 (95% CI: 0.536–0.957) and 0.602 (95% CI: 0.311–0.899) for training and validation cohorts, separately. Meanwhile, its AUC, ACC, PPV, NPV, sensitivity, and specificity were 0.75, 77%, 71%, 80%, 44%, and 72% separately, as well as 0.69, 70%, 64%, 68%, 42%, and 70%, respectively, for training and validation cohorts (Table 2). Later, the radiomics-based nomogram was also constructed by integrating radiomics signature (*P* < .001) and every independent clinical factor, and its C-index values were 0.912 and 0.863 for training and validation cohorts, separately (Fig. 2). Its AUC, ACC, PPV, NPV, sensitivity, and specificity were 0.95, 96%, 95%, 91%, 89%, and 86%, respectively, and 0.89, 85%, 94%, 88%, 86%, and 89%, respectively, for training and validation cohorts, separately (Table 2). As observed from the calibration curves, the actual LTP probabilities predicted by radiomics nomogram were consistent with those predicted by the nomogram for both cohorts, as observed from Figure 3. Figure 2 shows decision curve analysis of radiomics nomogram, suggesting that our as-constructed radiomics nomogram performed well. The preoperative radiomics-based nomogram yielded the best discriminatory ability, with C-index values of 0.84 (95% CI: 0.80–0.89) in the training cohort and 0.82 (95% CI: 0.77–0.88) in the validation cohort.

### 3.4. Significance of radiomics signature in survival prediction

According to survival analysis, LTP after MWA showed markedly dismal PFS compared with non-LTP (3-year PFS 42.6% vs 78.4%, *P* < .01). Moreover, significant radscore was selected by log-rank test, typically, radscore of −0.289 was applied in classifying patients as low- or high-risk group of different PFS. Low-risk patients with higher radiomics signature showed markedly superior PFS to higher-risk patients (3-year PFS 80.2% vs 48.6%, *P* < .01).

## 4. Discussion

This work focused on developing and validating models predicting local recurrence risk on the basis of pre-MWA contrast-enhanced CT radiomics in recurrent CRLM patients following hepatic resection. According to our results, incorporating radiomic features in the recurrence prediction model showed better ability in predicting recurrent CRLM. Specifically, the radiomics-based model outperformed others in prognosis prediction (C-index > 0.80; *P* < .05) and had better clinical usefulness, with well model calibration. Additionally, the model performed well in classifying CRLM to 2 recurrence-risk subgroups. Besides, this radiomics-based model had close performances in both cohorts, suggesting that it was reproducible and reliable. In this work, radiomics could be used to identify some prognostic markers affecting treatment option among CRLM patients, thereby obtaining the more individualized method. Actually, radiomics parameters may be correlated with LTP, which is advantageous relative to qualitative imaging assessment, in that it allows for tailoring tumor treatment, predicting thermal ablation therapeutic response, distinguishing patients with good prognosis from those with dismal prognostic outcome, and selecting candidate patients for MWA treatment. These results show that radiomics has better prediction accuracy and specificity than traditional clinical indicators. The combination of radiomics model with clinical indicators is beneficial to further optimize the prediction efficiency of the model. Under such circumstances, these findings verified that radiomics-based nomogram might be used to tailor tumor treatment for patients, estimate therapeutic response, select candidate patients with good prognosis from those with dismal prognosis, and choose candidate for MWA procedure.

Typically, the critical radiomics feature for model computation was histogram skewness on pre-ablation CT. The large negative skewness value stands for the asymmetric density distribution to high-densities associated with enhancement. Consequently, enhancement is probably related to nodular local aggressiveness, and previous works analyzed the metastasis shape and size as risk factors related to local recurrence.^[24]^ Volume was the second critical feature, conforming to prior discussion on longest diameter.^[24,25]^ Such result explained the obvious relation of image feature with nodule size. In addition, GLCM_Energy was associated with angular second moment, and it used for quantifying homogeneity. The energy level of heterogeneous regions decreases relative to homogeneous regions. This probably reflects the early ablation zone with non-distinguishable nodule in it, and it shows an increased local recurrence risk. Likewise, GLZLM_LZE, corresponding to long homogeneous zone distribution, was related to local recurrence and might reflect difficulties in nodule identification within dense ablation zones. CT-based radiomics analyses in other radiomics studies present promising outcomes in predicting CRLM local recurrence soon after ablation.^[17]^ Moreover, 1 study assessing if CT-based radiomics in ablation zone could be used to predict LTP following thermal ablation for CRLM also reported encouraging results.^[26]^ According to their results, a combined prediction model achieved markedly elevated accuracy compared with a clinical model.

In radiomics research, the use of machine learning methods to establish predictive models has gradually become an important part of the radiomics research process. Previous studies through combining radiomics with machine learning methods not only predict the status of microsatellite instability and lymph node metastasis of colorectal cancer, but also more accurately predict the curative effect of adjuvant chemotherapy and local treatment, as well as the prognosis of surgical resection.^[27,28]^ Previous studies have used specific machine learning approaches, like random forest, SVM, etc, and also included a variety of machine learning methods for comparison. These studies suggest that random forests and SVMs can be more accurately involved in the construction of diagnostic and prognostic models. In our study, SVM, supported in prior works, is used in classification and has achieved good results. Taghavi et al developed one machine learning radiomics model for predicting LTP according to CT scan in CRLM patients prior to ablation. This model achieved satisfactory prediction training and validation cohorts (C-indexes, 0.82 and 0.78), and was supposed as a valuable biomarker for prediction of LTP.^[17]^ However, Granata et al performed machine learning and radiomics analyses on the basis of MRI for assessing mucinous CRLM, which found k-nearest neighbor was inferior to the optimal linear regression model with regard to the precision.^[29]^

In this work, the radiomics-based model incorporating clinical indicators, including chemotherapy before liver MWA, TNM stage of primary tumor, maximum tumor diameter, and tumor number was constructed. The clinical indicators exhibited good predictive efficiency for the LTP of recurrent CRLM after MWA, especially when combined with radiomics models. Furthermore, TNM stage of primary tumor was positively correlated with recurrence. Nakanishi et al^[30]^ reported the radiomics-based model used to predict oxaliplatin-based chemotherapeutic response in CRLM. They selected TNM stage of primary tumor as risk factor for LTP. Some previous studies also have reported the value of TNM stage of primary tumor as predictor of recurrence for patients with CRLM.^[31,32]^ It has also been reported that chemotherapy after surgery was positively associated with tumor local progression. In addition, as mentioned in some research, periablational safety margin could be adopted for the independent prediction of LTP in CRLM, with ablations of safety margins >5 to 10 mm showing decreased LTP rates.^[33]^ According to our results, margin size significantly predicted LTP for MWA, which highlighted that it was important to achieve adequate ablation margins by MWA in the model. The larger tumor diameter has significantly higher local recurrence rate.^[34]^ The predictive value of tumor diameter with regard to LTP of CRLM is well established.

Certain limitations should be noted in this work. First, this was the retrospective study, which was associated with inherent biases, even though result reliability was improved through validation. Second, confirmatory biopsy was not obtained routinely, yet nodules showed unequivocal appearance and evolution without an infectious or inflammatory process. The MWAs were verified by one multidisciplinary oncological team. 18F-fluorodeoxyglucose positron emission tomography contributed to verifying nodule metastatic nature. Nonetheless, such technique is not sensitive in small nodules or specific with intermediate absorption. Third, we did not take genomic features into consideration. Recently, the association between prognosis and gene mutations in colorectal cancer was well known. Moreover, radiogenomics, focusing on relation of imaging phenotypes with genomics, emerges in cancer research and arouses wide attention. But it remains to be determined if simple construction of a model by applying imaging features is superior to radiogenomic analysis in outcome prediction.

In conclusion, this study presents a radiomics-based nomogram that incorporates clinical risk factors, and can be conveniently used to facilitate the preoperative individualized prediction of local recurrence in patients with recurrent CRLM following resection.

## Author contributions

**Conceptualization:** Bo Zhai, Hao Hu, Jia Chang Chi.

**Data curation:** Hao Hu, Jia Chang Chi.

**Formal analysis:** Hao Hu, Jia Chang Chi.

**Funding acquisition:** Hao Hu, Jia Chang Chi.

**Investigation:** Hao Hu.

**Methodology:** Hao Hu.

**Project administration:** Hao Hu, Jin He Guo.

**Resources:** Bo Zhai, Hao Hu, Jin He Guo.

**Software:** Hao Hu.

**Supervision:** Hao Hu.

**Validation:** Hao Hu.

## References

[1] Bray F Ferlay J Soerjomataram I. Global cancer statistics 2018: GLOBOCAN estimates of incidence and mortality worldwide for 36 cancers in 185 countries. CA Cancer J Clin. 2018;68:394–424. 30207593 10.3322/caac.21492

[2] Nordlinger B Sorbye H Glimelius B. Perioperative FOLFOX4 chemotherapy and surgery versus surgery alone for resectable liver metastases from colorectal cancer (EORTC 40983): long-term results of a randomised, controlled, phase 3 trial. Lancet Oncol. 2013;14:1208–15. 24120480 10.1016/S1470-2045(13)70447-9

[3] Syn NL Kabir T Koh YX. Survival advantage of laparoscopic versus open resection for colorectal liver metastases: a meta-analysis of individual patient data from randomized trials and propensity-score matched studies. Ann Surg. 2020;272:253–65. 32675538 10.1097/SLA.0000000000003672

[4] Zabaleta J Aguinagalde B Fuentes MG. Survival after lung metastasectomy for colorectal cancer: importance of previous liver metastasis as a prognostic factor. Eur J Surg Oncol. 2011;37:786–90. 21723689 10.1016/j.ejso.2011.05.014

[5] Jones NB McNally ME Malhotra L. Repeat hepatectomy for metastatic colorectal cancer is safe but marginally effective. Ann Surg Oncol. 2012;19:2224–9. 22207046 10.1245/s10434-011-2179-0

[6] Adam R Wicherts DA de Haas RJ. Patients with initially unresectable colorectal liver metastases: is there a possibility of cure? J Clin Oncol. 2009;27:1829–35. 19273699 10.1200/JCO.2008.19.9273

[7] Vasiniotis KN Kaye EA Sofocleous CT. Image-guided thermal ablation for colorectal liver metastases. Tech Vasc Interv Radiol. 2020;23:100672. 32591188 10.1016/j.tvir.2020.100672

[8] Gillams AR Lees WR. Five-year survival in 309 patients with colorectal liver metastases treated with radiofrequency ablation. Eur Radiol. 2009;19:1206–13. 19137310 10.1007/s00330-008-1258-5

[9] Di Martino M Rompianesi G Mora-Guzmán I. Systematic review and meta-analysis of local ablative therapies for resectable colorectal liver metastases. Eur J Surg Oncol. 2020;46:772–81. 31862133 10.1016/j.ejso.2019.12.003

[10] Han K Kim J Yang S. A single-center retrospective analysis of periprocedural variables affecting local tumor progression after radiofrequency ablation of colorectal cancer liver metastases. Radiology. 2021;298:212–8. 33170105 10.1148/radiol.2020200109

[11] Beamish P Lemke M Li J. Validation of clinical risk score for colorectal liver metastases resected in a contemporary multicenter cohort. HPB (Oxford). 2017;19:675–81. 28495435 10.1016/j.hpb.2017.03.010

[12] Benson AB Venook AP Al-Hawary MM. Colon cancer, version 22021, NCCN clinical practice guidelines in oncology. J Natl Compr Canc Netw. 2021;19:329–59. 33724754 10.6004/jnccn.2021.0012

[13] De Cobelli F Calandri M Della Corte A. Multi institutional analysis of outcomes for thermosphere microwave ablation treatment of colorectal liver metastases: the SMAC study. Eur Radiol. 2022;32:4147–59. 35092474 10.1007/s00330-021-08497-2 PMC9123066

[14] Ruers T Punt C Van Coevorden F. Radiofrequency ablation combined with systemic treatment versus systemic treatment alone in patients with non-resectable colorectal liver metastases: a randomized EORTC Intergroup phase II study (EORTC 40004). Ann Oncol. 2012;23:2619–26. 22431703 10.1093/annonc/mds053 PMC3457746

[15] Gillies RJ Kinahan PE Hricak H. Radiomics: images are more than pictures, they are data. Radiology. 2016;278:563–77. 26579733 10.1148/radiol.2015151169 PMC4734157

[16] Lambin P Leijenaar RT Deist TM. Radiomics: the bridge between medical imaging and personalized medicine. Nat Rev Clin Oncol. 2017;14:749–62. 28975929 10.1038/nrclinonc.2017.141

[17] Taghavi M Staal F Gomez Munoz F. CT-based radiomics analysis before thermal ablation to predict local tumor progression for colorectal liver metastases. Cardiovasc Intervent Radiol. 2021;44:913–20. 33506278 10.1007/s00270-020-02735-8

[18] Qin S Hu H Cui R. A prognostic nomogram for intrahepatic progression-free survival in patients with colorectal liver metastases after ultrasound-guided percutaneous microwave ablation. Int J Hyperthermia. 2022;39:144–54. 35012413 10.1080/02656736.2021.2023226

[19] Wang Y Zheng J Chen H. A prognostic nomogram for colorectal cancer liver metastases after percutaneous thermal ablation. Int J Hyperthermia. 2018;34:853–62. 28826279 10.1080/02656736.2017.1368095

[20] Ahmed M Solbiati L Brace CL. Image-guided tumor ablation: standardization of terminology and reporting criteria—a 10-year update. Radiology. 2014;273:241–60. 24927329 10.1148/radiol.14132958 PMC4263618

[21] Wang X Sofocleous CT Erinjeri JP. Margin size is an independent predictor of local tumor progression after ablation of colon cancer liver metastases. Cardiovasc Intervent Radiol. 2013;36:166–75. 22535243 10.1007/s00270-012-0377-1 PMC4122121

[22] Gillams A Goldberg N Ahmed M. Thermal ablation of colorectal liver metastases: a position paper by an international panel of ablation experts, the interventional oncology sans frontières meeting 2013. Eur Radiol. 2015;25:3438–54. 25994193 10.1007/s00330-015-3779-z PMC4636513

[23] Safi AF Kauke M Grandoch A. Volumetric Analysis of 700 Mandibular Condyles Based Upon Cone Beam Computed Tomography. J Craniofac Surg. 2018;29:506–9. 29215437 10.1097/SCS.0000000000004136

[24] Kelahan LC Kim D Soliman M. Role of hepatic metastatic lesion size on inter-reader reproducibility of CT-based radiomics features. Eur Radiol. 2022;32:4025–33. 35080646 10.1007/s00330-021-08526-0

[25] Maclean D Tsakok M Gleeson F. Comprehensive imaging characterization of colorectal liver metastases. Front Oncol. 2021;11:730854. 34950575 10.3389/fonc.2021.730854 PMC8688250

[26] Staal FCR Taghavi M van der Reijd DJ. Predicting local tumour progression after ablation for colorectal liver metastases: CT-based radiomics of the ablation zone. Eur J Radiol. 2021;141:109773. 34022475 10.1016/j.ejrad.2021.109773

[27] Rompianesi G Pegoraro F Ceresa CD. Artificial intelligence in the diagnosis and management of colorectal cancer liver metastases. World J Gastroenterol. 2022;28:108–22. 35125822 10.3748/wjg.v28.i1.108 PMC8793013

[28] Tabari A Chan SM Omar OMF. Role of machine learning in precision oncology: applications in gastrointestinal cancers. Cancers (Basel). 2022;15:63. 36612061 10.3390/cancers15010063 PMC9817513

[29] Granata V Fusco R De Muzio F. Radiomics and machine learning analysis based on magnetic resonance imaging in the assessment of liver mucinous colorectal metastases. Radiol Med. 2022;127:763–72. 35653011 10.1007/s11547-022-01501-9

[30] Nakanishi R Oki E Hasuda H. Radiomics texture analysis for the identification of colorectal liver metastases sensitive to first-line oxaliplatin-based chemotherapy. Ann Surg Oncol. 2021;28:2975–85. 33454878 10.1245/s10434-020-09581-5

[31] Wei S Han Y Zeng H. Radiomics diagnosed histopathological growth pattern in prediction of response and 1-year progression free survival for colorectal liver metastases patients treated with bevacizumab containing chemotherapy. Eur J Radiol. 2021;142:109863. 34343846 10.1016/j.ejrad.2021.109863

[32] Badic B Da-Ano R Poirot K. Prediction of recurrence after surgery in colorectal cancer patients using radiomics from diagnostic contrast-enhanced computed tomography: a two-center study. Eur Radiol. 2022;32:405–14. 34170367 10.1007/s00330-021-08104-4

[33] Shady W Petre EN Do KG. Percutaneous microwave versus radiofrequency ablation of colorectal liver metastases: ablation with clear margins (A0) provides the best local tumor control. J Vasc Interv Radiol. 2018;29:268–275.e1. 29203394 10.1016/j.jvir.2017.08.021 PMC5803367

[34] Vogl TJ Zitsch M Albrecht M. Radiofrequency versus microwave ablation for intraoperative treatment of colorectal liver metastases. Eur J Surg Oncol. 2022;48:834–40. 34686404 10.1016/j.ejso.2021.10.012

